# Supplementary material for: Co-Surveillance of Rotaviruses in Humans and Domestic Animals in Central Uganda Reveals Circulation of Wide Genotype Diversity in the Animals
Source: Viruses. 2023 Mar 13;15(3):738. doi: 10.3390/v15030738 (PMC10052166; doi:10.3390/v15030738)
Supplement: Supplementary file 1 [file viruses-15-00738-s001.zip › viruses-2267312-supplementary.pdf]

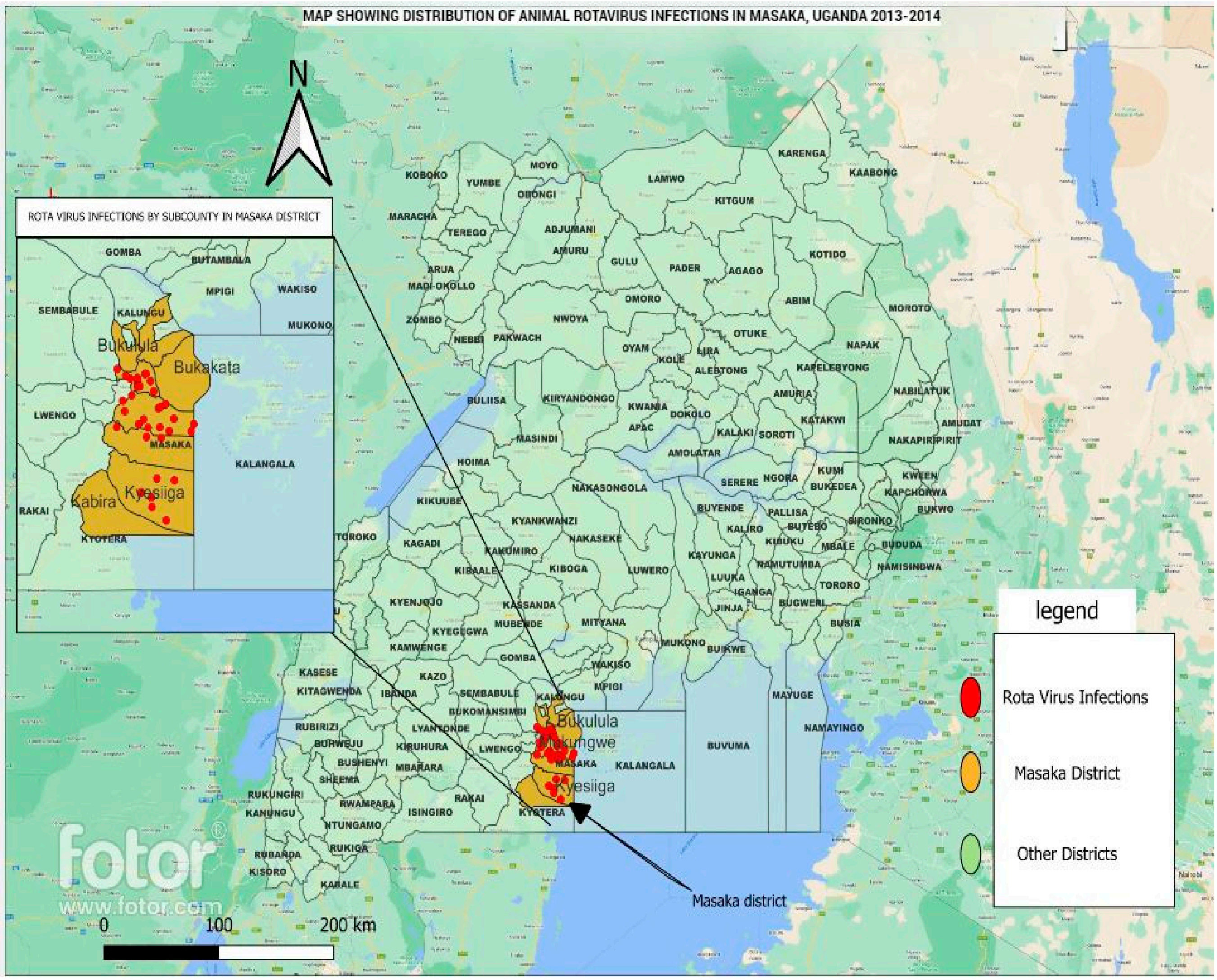

Supplementary Figure S1. Distribution of Animal Rotavirus infections in Masaka District, Uganda, 2013-2014.
